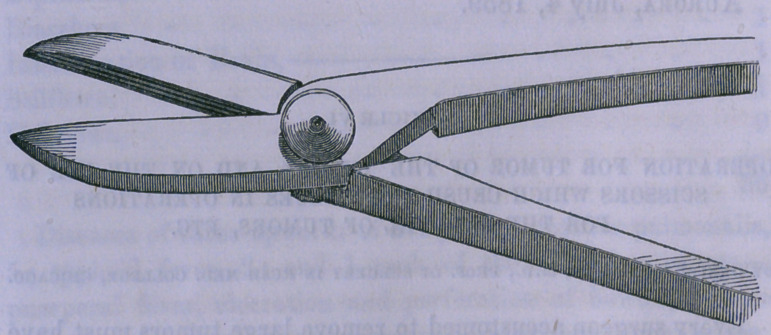# Operation for Tumor of the Throat, and on the Use of Scissors Which Crush the Tissues in Operations for the Removal of Tumors, Etc.

**Published:** 1859-08

**Authors:** Daniel Brainard

**Affiliations:** Prof. of Surgery in Rush Med. College, Chicago


ARTICLE VI.

### OPERATION FOR TUMOR OF THE THROAT, AND ON THE USE OF SCISSORS WHICH CRUSH THE TISSUES IN OPERATIONS FOR THE REMOVAL OF TUMORS, ETC.

BY DANIEL BRAINARD, M.D., PROF. OF SURGERY IN RUSH MED. COLLEGE, CHICAGO.

Every surgeon accustomed to remove large tumors must have found the advantage, indeed the necessity at times, of resorting to laceration of the tissues with the finger, instead of dividing them out with the knife. The benefits derived from the adoption of this course are greater rapidity, diminished hemorrhage, and consequently greater safety. Many tumors have been successfully removed in this way, which otherwise would have been beyond the reach of surgery.

M. Chassaignac, by his instrument called *ecraseur liniere*, has rendered possible the application of the process of laceration to a class of cases in which it had before been impossible; and in operations such as those for polypi, hemorrhoidal, vascular and pediculated tumors of large size, nothing more perfect than his instrument seems desirable. But in many cases to which M. Chassaignac has applied it, such as cancer of the rectum, of the female breast, etc., and in others still in which its application is impossible, such as deep-seated tumors and certain forms of nasal polypi, it has seemed to me that an instrument acting upon the same principle, and capable of being more readily applied, might have advantages over it.

I accordingly have had constructed a forceps somewhat similar to the bone scissors of Liston, with which, pieces of tissue, no matter how firm (if not bony) or deeply situated, can be readily seized and crushed, so as to save time and avoid the danger of hemorrhage.

The figure of this instrument, half size, is given below. It is likely that a skilful instrument maker could improve upon the form of it; the principle is what I desire particularly to call the attention of surgeons to.

That division by a blunt instrument has, in some cases, advantages over incision is well known, but these advantages are as yet not fully appreciated. Pain having been annulled by the use of chloroform, it only remains to avoid bleeding, the shock of operations, and absorption of pus and other deleterious fluids, and many terrible operations will be shorn of their terrors. It is believed that a more general application of the crushing process will tend materially to this end. It is with this hope that this instrument is submitted to the profession.

The first case in which it was used was that of Joseph Brown, from Wisconsin, operated June 2, 1859. The subject was a healthy man, 31 years of age, having a tumor of the size of an orange filling up the back part of the mouth. He stated that this had existed for twenty years, but was of small size until within the last two years, during which time it has grown rapidly, and at present interferes materially with deglutition and respiration.

On looking into the mouth, the whole of the back part is found filled with this growth, which extends from the right to the left side, and from the middle of the roof of the mouth to the base of the tongue. The surface is of a deep red color; form lobulated; to the touch, elastic, but not fluctuating. Has never been painful. On puncture with an exploring needle, bleeds freely. This growth, according to the recollections of the patient, seemed to have originated in the veil of the palate, a little nearer to the left than to the right side.

On attempting to dissect it out, the bleeding from a small incision was so free as to give much trouble, and even render the propriety of attempting it doubtful. It was then that the crushing forceps were resorted to. The tissue of the roof of the mouth being raised in successive portions, by passing under each an aneurismal needle sharpened, these were crushed and divided without the occurrence of any troublesome hemorrhage. The same process was then used at the sides. After the connections had been in a measure loosened, a large-sized curved needle, armed with a strong ligature, was passed through it. This enabled me to draw it forward so that the remaining attachments could be partly divided with the finger. The process of separation was completed with the crushing forceps. The posterior layer of the veil of the palate was now divided. This contracted, and at the end of three days the uvula, which had been depressed beyond the reach of the finger, appeared in near its natural position.

The tumor, on cutting through it, presented the appearance of what is called *adenoid* by Velpeau, in his work on the diseases of the female breast. It was firm, uniform throughout in appearance, except at one point where there was a small cavity containing reddish serum. It was surrounded on all sides by a firm fibrous covering, which also extended between the different divisions. This, and the length of time during which the tumor had existed, make me believe that it is not malignant.

Its removal would have been very difficult without the forceps, as the knife allowed too much bleeding and the *ecraseur* could scarcely be applied.

No accident occurred, and the patient was able to return home at the end of two weeks, with the wound nearly cicatrized.

## Figures and Tables

**Figure f1:**